# Case of Disease of the Bones and Joints of the Lower Extremity;—Spontaneous Dislocation of the Hip Joint;—Amputation;—Discharge of Calculi from the Bladder;—Recovery

**Published:** 1845-01

**Authors:** William Monro

**Affiliations:** Surgeon to the Dundee Infirmary


*Case of Disease of the Bones and Joints of the Lower Extremity ;—Spontaneous Dislocation of the Hip Joint ;—Amputation ;—Discharge of Calculi from the Bladder ;—Recovery.* By William Monro, M. D., Surgeon to the Dundee Infirmary.

*Jan.* 2,1841.—George Nicol, aged 6, fell when engaged in play, upon his left knee. He did not complain much of the part for two days, when swelling appeared on its anterior surface, extending down the limb,—confining him to bed, with inability to move. The swelling then increased, attended by great constitutional disturbance, and heat and redness, extending as far as the ankle, but being most considerable over the upper third of the anterior aspect of the tibia. Leeches and aperients were ordered, and repeated for a few days. An incision was then made about two inches below the head of the tibia, which gave exit to a considerable quantity of unhealthy purulent matter. The bone was bare. For several days the patient improved; but subsequently, the inflammation attacked the knee-joint, with suppuration and ulceration of the cartilages; great thickening of the femur itself, followed by openings on each side of the knee-joint, which discharged unhealthy pus, and through which the bone could be felt bare. At this time, the tibia became exposed throughout its whole length, without any attempt at reproduction.

About the first of February, he complained of pain in the right hip, which for some days was attributed to pressure from lying upon it. On the fourth, however, after more careful examination, it was found to be much swollen, red and painful directly over, and for some extent around the head of the femur. Warm poultices and fomentations were applied; and in a few days fluctuation was detected. On the 13th a small opening was made in the most prominent part, by which was discharged about three ounces of unhealthy curdy pus. The wound continued to discharge for a considerable time, but the swelling and pain greatly diminished.

*March* 2.—On consultation with Dr. Gibson, the head of the femur was found completely luxated, and drawn upwards and backwards on the dorsum of the ileum; the discharge was now moderate, and the swelling nearly gone. Pressure over the joint was made by means of strips of adhesive plaster, a compress and bandage; and in the course of three weeks the sinus healed. Ultimately the swelling and pain entirely disappeared, and nothing remained except the complete dislocation.

It may be scarcely necessary to remark, that during this long and severe illness, the boy's general health had suffered greatly. He became extremely emaciated, had a quick pulse, night sweats, occasional diarrhœa, loss of appetite, and all the usual symptoms of hectic fever. Care was taken to regulate the bowels by diet and medicine. The body was frequently sponged with vinegar and water, with great comfort and advantage. His diet had been chiefly farinaceous, with, occasionally, light soups; but wine and malt liquors have never been found to agree with him. Quinine, also, was several times tried, but without advantage; small doses of Dover's powder, with hydr. cum cretâ, and rhubarb, were frequently administered, with the best possible result.

*June* 1.—The fever has considerably abated; the appetite is improved; the bowels less relaxed; the skin softer; the tongue moist, but reddish at the tip and edges. The right hip is free from pain and swelling; its motions are greatly impaired, but the joint is not anchylosed; the left knee is discharging unhealthy matter from several openings in both its inner and outer sides; there is also an opening about three inches above the joint on the inside of the thigh, through all of which diseased bone can be easily detected by the probe. Several openings over the tibia continue to discharge a thin unhealthy matter, and that bone can be detected quite denuded of its periosteum throughout nearly its whole extent.

Having at this time consulted with Dr. Gibson and Mr. Kirkland, it was agreed that the left limb should be removed at the upper third of the thigh. This I accordingly did on the 7th, when I had the assistance of my friends above named. In consequence of the contraction of the knee-joint, the flexion of the leg, and the impossibility of extending it, I was obliged to take the flaps from the anterior and posterior surfaces of the thigh. They answered extremely well, forming an excellent cushion over the end of the bone. There was nothing remarkable in the operation. Three vessels required ligatures; very little blood was lost. He stood the operation well. The wound was cleaned, and the edges immediately brought together, and retained by four stitches, strips of adhesive plaster, and bandages.

On examining the amputated limb, we found the whole tibia to consist of a soft brownish dirty-looking gelatinous substance, having a thin covering of dead bony matter, resembling, in consistence and thickness, the shell of an egg, with some lamellæ of bone passing through the gelatinous matter, the whole so soft that a probe could be easily pushed through it in any direction; indeed, had it not been for the soundness of the fibula, the limb could not have preserved its straight form; about three inches below the knee, an oblique fracture of the bone, if it could be called so, had taken place; this, however, I am satisfied, was only the result of the moving of the limb during the progress of the disease, and not the effect of the primary accident; there was, however, very little displacement. The heads of the tibia, fibula, the lower extremity of the femur, and the patella were so soft and spongy, that a knife could be easily thrust through them; for about two inches above the condyles, the femur had the same soft sponginess; above this, for some way, the periosteum was much thickened, and the cellular tissue infiltrated with lymph, which had assumed a firm fibrous appearance; the muscular tissue was pale and much wasted. The cartilages were ulcerated, and the joint communicated freely in all directions with the sinuses which existed around it. The epiphyses of the femur and tibia were loose, and almost separated from the shafts of these bones.

For two days the patient went on well. On the 10th June he complained of difficulty of voiding his urine, for which he had an anodyne, castor oil, and warm fomentations, with relief. On the 11th, however, he had complete retention, and on examination, I found a calculus in the urethra, about four inches from its extremity, completely obstructing the passage; by gentle pressure it was brought near to the extremity, and then scooped out. On the 12th the stump was dressed. Union by the first intention had taken place throughout the greater part of the wound. In the situation of the ligatures there was a considerable discharge of healthy pus. On the 15th (when one of the ligatures came away,) the wound was looking well, and the boy's health improving. On the 17th another ligature came away, and every thing was advancing favourably; he had some appetite for food, slept well at night, and appeared quite cheerful. His back, which had been much excoriated by being kept moist from the constant discharge of urine for several days, was much better, with superficial ulceration, looking healthy. He voided urine freely, and without pain. His bowels were regular. On the 18th, he had sickness and vomiting of bilious looking-matter; was feverish, and without appetite. He was ordered to take at night, three grains of calomel, with two of Dover's powder.

*June* 19.—He was much better. His bowels were three times moved. The tongue was clean, and rather reddish. The stump looked well. The discharge was much less. About this time, he was raised frequently to the sitting posture, which he had not been for several months before, when an immense number of small calculi were discharged each time he voided his urine. I obtained about three dozen of them, varing from 1 to 3 grains each. I sent a number of them to my friend Mr. Goodsir, of Edinburgh, to have them analysed for me; and, in the mean time under the impression that they consisted chiefly of lithic acid, I put the patient under the liquor potassæ—prescribing a dose three times a day.

*June* 22.—The remaining ligature on the femoral artery came away. He still passed numerous calculi from the urethra. His general health was improved.

*July* 8.—He has had some slight feverish attacks since last report; and has passed several rather large stones with difficulty and pain. The stump is quite whole.

*July* 25.—He had complete retention of urine, from a calculus sticking in the urethra, about an inch from the extremity. It was removed by the scooped extremity of a director, with immediate relief.

*Sept.* 1.—He has been occasionally distressed from the passing of large sized calculi, some weighing six grains. The magnesia solution was substituted for the aqua potassæ.

*Sept.* 22.—He was very ill all night from a large calculus becoming impacted about the membranous portion of the urethra. After considerable difficulty I succeeded in breaking it into pieces, by means of Weis's instrument, after which it passed readily. For some time past his general health has not been so good. His bowels were occasionally relaxed, with loss of appetite, which I suspect to have been in a great degree owing to indulgence in fruit and indigestible food. He has had frequently small doses of Dover's powder, rhubarb, and hydr. c. cretâ.

*Oct.* 1.—The patient having long been accustomed to void his urine in the recumbent position, and finding that he still continues this practice, I have insisted upon his always being raised erect, and even bent forward when emptying the bladder. The general health is improving, and he is gaining flesh. The stump is very sound and firm, having a good soft cushion over the end of bone. The right hip is entirely free from pain or swelling, the head of the femur can be very distinctly felt lying on the dorsum of the ilium, which, however, admits of considerable motion.

*Dec.* 1.—Since last report my patient continued gradually to gain health and strength, the calculi diminishing both in size and number, aud he has now passed none for several weeks, he has no complaint whatever, excepting the inability of using his right leg, which is stiff and weak.

From the above date, up to the present time, he has continued free from any disease, his urinary organs are completely sound, he has gained his former health and strength, and he is able to walk with the assistance of crutches.

11*th July*, 1844.—Since the above was written, the boy has continued in the enjoyment of excellent health, and is now able to use the crutches with great freedom, going and returning from school every day without any other aid.

On the 21st of February last I was favored with an analysis of the calculi by Dr. Wilson, of Edinburgh, to whom, and Mr. Goodsir, I beg to express my obligation; the analysis is so interesting, and the composition of the stones so unusual, that I think it proper to give Dr. Wilson's account of them in his own words.

*Analysis of the Calculi.*—By Dr. G. Wilson.—"The calculous matter sent me for examination was found on analysis to contain the following substances:

Carbonate of lime,
Carbonate of magnesia,
Phosphate of lime,
Muriate of ammonia, and
Animal matter.

"The substances are arranged in the order of their abundance, carbonate of lime being the chief ingredient; but as the quantity of matter I had to operate on was small, I could not ascertain with exactness the relative proportions of each.

"The presence of animal matter is indicated by a transient charring and blackening before the blow-pipe, accompanied with the evolution of a peculiar and characteristic animal odour.

"When the calculous matter is digested in distilled water, and the liquid filtered, muriate of ammonia is easily detected in the solution; caustic potass evolves ammonia from the calculi, but the quantity is very small.

"The calculi dissolve entirely, and with lively effervescence in warm nitric or muriatic acid. Acetic acid dissolves only the carbonates, leaving untouched the phosphate which can be identified in the usual way. Oxalate of ammonia, employed with the usual precautions, precipitated the lime, leaving the magnesia in solution.

"Lithic acid was carefully sought for, but not could be detected.

"Calculi containing the carbonate of lime and magnesia are mentioned by Dr. Kane as occasionally occurring. Dr. Prout has seen some rare cases, where carb. of lime was the chief ingredient; Dr. Yellowly has found this carbonate present in a great number of phosphatic calculi; Prout, Faraday and Brande, had done so likewise. The muriate of ammonia, though not generally included amongst the ingredient of calculi, has been shown by Brande and Yellowly, to be almost invariably present, but in small quantity."—*Ibid.*

